# Discharge with a smartphone application for follow-up after day care surgery: a randomised controlled trial

**DOI:** 10.1016/j.bjao.2025.100489

**Published:** 2025-09-25

**Authors:** Bram Thiel, Marc Godfried, Maaike van Emst, Lisette Vernooij, Liesbeth van Vliet, Eva Rumke, Marc Snoeck, Seppe Koopman, Cor Kalkman

**Affiliations:** 1 Department of Anaesthesiology, OLVG Hospital, Amsterdam, the Netherlands; 2 Department of Anaesthesiology, Amsterdam University Medical Center, Amsterdam, the Netherlands; 3 Department of Intensive Care Medicine and Anesthesiology, University Medical Center Utrecht, Utrecht, the Netherlands; 4 Faculty of Behavioral Sciences, Leiden University, Leiden, the Netherlands; 5 Royal Military School Airforce, Dutch Ministry of Defence, Woensdrecht, the Netherlands; 6 Department of Anaesthesiology, CWZ Hospital Nijmegen, Nijmegen, the Netherlands; 7 Department of Anaesthesiology, Maasstad Hospital, Rotterdam, the Netherlands

**Keywords:** day care surgery, postoperative pain and nausea, quality of recovery, self-recording, smartphone application

## Abstract

**Background:**

Day care surgery patients have limited options to communicate pain or nausea to their healthcare providers after discharge. This study evaluated the effectiveness of a smartphone application for pain and nausea follow-up as an enhancement to standard care.

**Methods:**

We performed a multi center non-blinded prospective randomised controlled trial including day care surgery patients. The intervention group received a smartphone application with bidirectional messaging and standard care for postoperative follow-up. The control group received standard care. The primary outcome was measured with the Quality of Recovery-15 scale on the seventh postoperative day. Secondary endpoints included quality of recovery at postoperative day 1 and 4, satisfaction with care, trust in hospital care, and patient remarks concerning recovery and complications.

**Results:**

Out of 301 included patients, 149 were randomly assigned to the smartphone application and 152 to standard care. Perioperative characteristics were comparable between the groups. On postoperative day 7, no difference was observed in quality of recovery, with median difference 0.0 (95% confidence interval: −10.0 to 7.0; *P*=0.56). Ratings of satisfaction, trust, and to recommend the hospital showed no clinically important differences.

**Conclusions:**

We found no differences in patient-reported quality of recovery for day care surgical patients when adding postoperative follow-up of pain and nausea via a smartphone application compared with standard care alone.

**Clinical trial registration:**

NCT05244772

**Editor’s key points**

• In this prospective, multicenter, non-randomized Dutch trial, the addition of a smartphone application for postoperative follow-up did not improve quality of recovery compared with standard care alone in day-case surgical patients. Future studies evaluating the impact of postoperative smartphone application use on patient-centered outcomes should be rigorously designed and conducted to guide evidence-based best practices.

The volume and complexity of surgical procedures have increased with a wider range of patient populations now considered suitable for day care surgery, for example, same-day knee joint replacement.^1^ Currently, most patients undergoing minor to intermediate procedures are discharged within 4–5 h.^2^^,^^3^ After discharge, patients receive verbal and written instructions on managing pain, nausea, and potential surgical complications. However, up to 75% of patients report moderate to severe postoperative pain and 28% to 57% experience nausea or vomiting.^4^, ^5^, ^6^, ^7^, ^8^, ^9^ Communication barriers, including voicemail systems and limited staff availability, complicate patient access to support. An accessible eHealth tool could support postoperative care by enabling bidirectional communication between patients and healthcare professionals. EHealth tools are already used in perioperative care for monitoring, education, and tele-rehabilitation, offering patients reliable and approachable resources.^10^^,^^11^ Despite promising early results with a similar application amongst clinically admitted patients,^12^ the effects on recovery after day care surgery are unknown.

We hypothesised that enabling self-recording with a smartphone application for postoperative follow-up with empathetic feedback from healthcare professionals in addition to the standard care improves patient-experienced quality of recovery. The primary objective was to compare perceived quality of recovery, assessed by the Quality of Recovery-15 (QoR-15) scale,^13^^,^^14^ on postoperative day 7 between patients with standard care with the smartphone application and those receiving standard care only.

## Methods

### Study design and setting

We conducted a prospective non-blinded randomised controlled trial across three Dutch general hospitals: Onze Lieve Vrouwe Gasthuis Hospital (OLVG), Amsterdam; Maasstad Hospital (MSH), Rotterdam; and Canisius Wilhelmina Hospital (CWH), Nijmegen. The trial was approved (23 February 2022) by the Medical Ethics Committees United (MEC-U) with registration number R21.076/NL78144.100.21 and registered at the ClinicalTrials.gov public website (16 February 2022, number NCT05244772). The study protocol has been published previously.^15^ Manuscript preparation adhered to the Consolidated Standards of Reporting Trials (CONSORT) guidelines and relevant extensions.^16^^,^^17^

### Participants

Eligible patients were those scheduled for elective day care surgery aged ≥18 yr with ASA classification 1–3 and in possession of a smartphone with iOS or Android operating system. Patients who were non-Dutch speakers, had intellectual disability, or were not able to install the application were excluded. Patients needing unanticipated overnight hospital stay were replaced.

### Intervention

Perioperative anaesthesia standard care was provided according to the guidelines of the Dutch Association of Anaesthesiologists (NVA).^18^^,^^19^

Patients were asked about their pain and nausea at least once per perioperative phase (e.g. recovery room, day care ward). Pain management included paracetamol (acetaminophen), non-steroidal inflammatory drugs (NSAIDs), or opioids. In case of nausea, metoclopramide or a 5HT3-antagonist was administered. Before discharge, all patients received verbal and paper instructions from the day care ward nurse. In addition, they received a medication box containing paracetamol, NSAID, metoclopramide, or a proton pump inhibitor (if indicated) and opioids (if indicated) for 4 days maximum.

In addition to standard care, the smartphone group was instructed to install the smartphone application to self-record pain and nausea and communicate with healthcare providers via a secure platform after discharge. Patients could report pain and nausea at any time and received a daily push notification at 10:00 with the question if they were in pain or nauseous. The application was operational for 7 days post-discharge. For pain, patients answered questions about its severity, impact, and need for intervention, rating it on a numerical scale (0–10). For nausea, Myles’ Postoperative Nausea and Vomiting (PONV) scale was used to assess its impact, associated symptoms, and medication use.^20^ A comment section allowed for additional input. Trigger alerts were set for the in-app questions ‘do you want something done to relieve your pain or nausea?’ or an increase of three points on the numerical rating scale (NRS) for pain and additional questions asked in the app’s messaging service. In case of an alert or question, an anaesthesia medical assistant was available 7 days a week from 8:00 to 16:30 to respond by using the application’s messaging service or by telephone. Patients who sent a request via the app in the evening or at night received a response the following day. Telephone assessment followed a protocolised evaluation of pain, nausea, and physical status. In case of a medical alert, the medical assistant consulted the anaesthesiologist, nurse specialist, or physician assistant for appropriate intervention.

The smartphone application is a reconfiguration of an already existing and clinically used monitoring platform by Luscii Healthtech B.V., Amsterdam, the Netherlands.^21^^,^^22^ The configuration for postoperative monitoring was commissioned by OLVG Hospital, Amsterdam. During this study, versions 2.43.0 or higher were used. The application is CE class IIa certified.^23^^,^^24^

### Outcomes

The primary outcome was patient-reported quality of recovery measured with the QoR-15 scale^14^ on day 7 after surgery. The QoR-15 scale is a validated 15-item scale for postoperative follow-up over the past 24 h assessing sleep, eating, sense of control, pain, nausea, and emotional well-being. Each item is scored on a scale from 0 (worst) to 10 (best). All patients were asked to complete the QoR-15 scale 1 day before admission (baseline score), and on the first, fourth, and seventh day post discharge. To maintain consistency with the validated use of the QoR-15 and to avoid selective outcome reporting, we based our main analyses on the total score. Furthermore, all patients were asked about the number of consultations with the hospital, general practitioner, or emergency department, and the number of surgical complications as evaluated using the Clavien–Dindo classification.^25^^,^^26^

Readmissions were defined as hospitalisations related to the surgical intervention. Patient satisfaction was measured using a visual analogue scale (‘not at all’ to ‘very much’, 0–10 range).^27^

Patients’ evaluations of their experienced care during the seventh day discharge period were assessed with two questions: (1) How likely is it that you would recommend this hospital to other day care surgery patients?, using an adapted item from the Consumer Quality Index (CQindex) (‘would definitely not recommend’ to ‘would definitely recommend’, 0–10 range).^28^^,^^29^ (2) What is your overall rating of the quality of care provided by the hospital during the 7-day discharge period?, using an item from the CQ index (‘very poor care’ to extremely good care’, 0–10 range).^28^^,^^29^

Data collection for the QoR-15 primary and secondary endpoints was conducted using Castor EDC (Amsterdam, the Netherlands), a digital data capture platform. Patients in both groups received an automated e-mail request to complete the questionnaire, which could be accessed via a URL link.

### Other study parameters

Preoperative patient characteristics included age, sex, smoking status, body mass index, ASA classification, pain NRS, history of (chronic) pain, history of motion sickness, previous surgical- or anaesthesia-related complications, and socioeconomic status (SES) (a measure of education, income, and occupation; 0 represents the Dutch national average).

Intra- and postoperative variables included surgical specialism, surgical procedure and risk classification, anaesthesia technique, PONV prophylaxis, surgery duration, intraoperative medication, pain NRS and PONV at recovery ward, pain NRS and PONV at day care ward, in-hospital surgical and anaesthesia complications, and duration of admission.

### Randomisation

Patients were randomly allocated to the smartphone group (intervention) or the standard care group (control). Treatment assignments were performed centrally according to a computer-generated random schedule (Castor EDC, 2022)^30^ in permuted blocks of four stratified per participating hospital. After randomisation, a study identification number was allocated to all included patients.

### Statistical plan and analysis

Sample size calculation was based on an 8.0-point difference in the overall score in the QoR-15 scale on postoperative day 7 considered as a minimal clinically relevant difference with standard deviation of 19.1.^31^ This led to a sample size of 91 patients per group to achieve 80% power, with a significance level (alpha) of 0.05 using a two-sided two-sample *t*-test. We anticipated for 20% dropout. An initial analysis limited to questionnaire response rates, excluding any analysis of the QoR-15 endpoint, was conducted after enrolment of 200 out of the first 203 patients, revealing that only 65% of participants had completed the questionnaires. Despite repeated e-mail reminders, even with the additional 20% patients accounted for dropout and incomplete data, an insufficient number of patients with complete endpoint data would be included. To address this, we increased the initial calculated sample size from 230 to 310 patients after approval of the MEC-U.

Analyses followed the intention-to-treat principle,^32^ excluding cases lost to follow-up, withdrawal of consent, or cancellation of surgery. Because many patients did not complete the QoR questionnaire on the specific follow-up days we had initially defined, we decided to group the completed questionnaires into broader time clusters. Questionnaires completed before surgery were categorised as baseline. Those completed on postoperative days 1, 2, or 3 were grouped under postoperative day 1 (POD1). Questionnaires filled in on days 4, 5, or 6 were categorised as postoperative day 4 (POD4). Finally, any questionnaires completed between day 7 and 14 after surgery were grouped under postoperative day 7 (POD7).

All outcomes were studied using descriptive statistics and compared with Mann–Whitney *U* test and χ^2^ when appropriate. Differences between groups were presented with estimated median differences or absolute differences in proportions with their accompanying 95% confidence interval (95% CI) using bootstrap percentile method (1000 replications) as most variables were non-normally distributed.

Data collection for the QoR-15 primary and secondary endpoints was conducted using Castor EDC, a digital data capture platform. Patients in both groups received an automated e-mail request to complete the questionnaire, which could be accessed via a URL link. Analyses were performed using IBM SPSS Statistics for Windows, Version 22.0 (IBM Corp., Armonk, NY, USA) and R statistics for Windows version 4.3.0 (The R Foundation for Statistical Computing, Vienna, Austria) for Windows. Data storage and validation were performed using an electronic Case Report Form in Castor EDC.^30^

## Results

**Fig 1:**
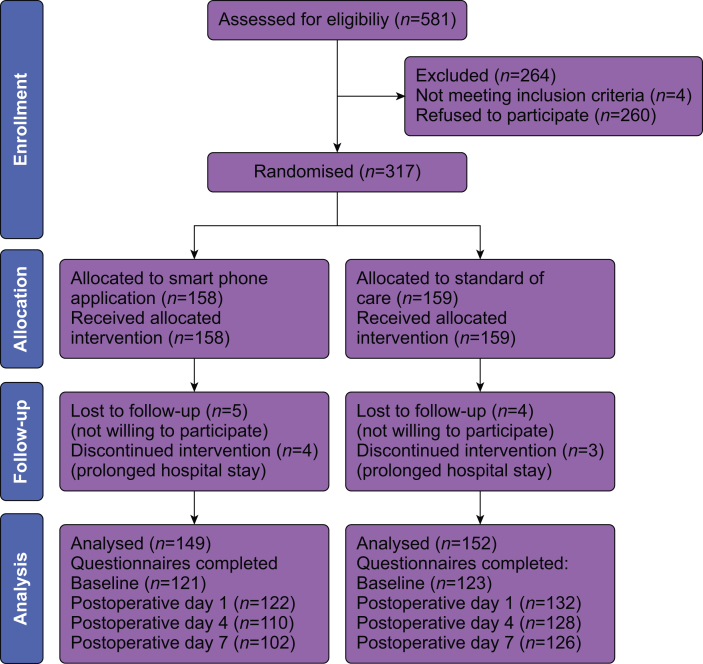
Consort patient flow diagram.

Patients were recruited between August 2022 and February 2023. In total, 301 patients were included in the analysis (Fig. 1). Of these, 149 were included in the smartphone group (intervention) and 152 in the standard care group (control). For primary outcome analysis, 199 patients completed both baseline and postoperative day 7 QoR-15 scale, 92 in the smartphone group and 107 in the standard care group. Characteristics were comparable between the groups except for the distribution of trauma surgery patients and the prescription of NSAIDs at discharge (Table 1). The smartphone application was used by 135 out of 149 patients in the intervention group, with an average usage of once per day. The medical assistant responded to all queries and all after-hours queries were replied to on the next day.

**Table 1:** Pre-, intra-, and postoperative characteristics.

	Smartphone application	Standard care
	(*n*=149)	(*n*=152)
Preoperative characteristics		
Sex, female, *n* (%)	84 (56)	84 (55)
Age (yr), median (IQR)	43 (31–57)	45 (32–59)
BMI (kg m^−2^), median (IQR)	26 (23–29)	25 (23–29)
Socioeconomic status, mean (sd)	−0.050 (0.245)	−0.039 (0.252)
Previous anaesthesia side-effects, *n* (%)	14 (9)	10 (7)
Preoperative use of analgesics, *n* (%)	33 (22)	28 (18)
QoR-15 baseline, median (IQR)	122 (105–137) (*n*=121)	119 (100–136) (*n*=123)
Intraoperative characteristics		
Surgical procedure, *n* (%)		
Ear, nose, throat surgery	33 (22)	33 (21)
General surgery	51 (34)	53 (35)
Gynaecological surgery	6 (4)	4 (3)
Orthopaedic surgery	23 (15)	17 (11)
Plastic surgery	7 (5)	7 (5)
Trauma surgery	27 (18)	37 (24)
Urogenital surgery	1 (1)	1 (1)
Vascular surgery	1 (1)	0
Risk of surgery∗, *n* (%)		
Minor risk surgery	135 (91)	131 (86)
Intermediate risk surgery	14 (14)	21 (14)
ASA physical status, *n* (%)		
ASA class 1	78 (52)	76 (50)
ASA class 2	65 (44)	69 (45)
ASA class 3	6 (4)	6 (4)
Anaesthesia technique, *n* (%)		
General anaesthesia	98 (66)	98 (65)
General—with local regional anaesthesia	18 (12)	18 (12)
Loco regional anaesthesia	23 (15)	27 (18)
Spinal anaesthesia	9 (6)	8 (5)
Spinal—with local anaesthesia	1 (1)	1 (1)
PONV prophylaxis	92 (62)	96 (63)
Postoperative pain and nausea during admission†, *n* (%)		
No pain (NRS 0)	69 (46)	70 (46)
Minor (NRS 1–4)	62 (42)	70 (46)
Intermediate (NRS 5–7)	17 (11)	11 (7)
Severe pain (NRS 8–10)	0	1 (1)
PONV	7 (5)	9 (6)
Prescribed discharge medication for use at home, *n* (%)		
Paracetamol (acetaminophen)	147 (99)	150 (99)
Non-steroidal anti-inflammatory drug	140 (94)	132 (87)
Tramadol	23 (15)	24 (16)
Oxycodone	62 (42)	64 (42)
Proton pump inhibitor	98 (66)	91 (60)
Antiemetic	70 (47)	66 43)

IQR, interquartile range; NRS, numerical rating scale; PONV, postoperative nausea and vomiting; QoR-15, Quality of Recovery-15.

∗ Risk of surgery, minor (e.g. breast, dental) or intermediate (e.g. head and neck, major orthopaedic, intraperitoneal).

† Highest NRS for pain, nurse assessed and recorded during postoperative recovery in hospital.

### Primary outcome

There was no difference in QoR-15 scale between the smartphone group, median 124 (interquartile range [IQR]: 107–137), and the standard care group, median 124 (IQR: 104–137) (median difference 0.0 [95% CI: −10 to 7.0]; *P*=0.56) (Table 2).

**Table 2:** Primary and secondary outcomes for POD1, POD4, and POD7.

	Smartphone application	Standard care	Difference (95% CI)	*P*
	(*n*=149)	(*n*=152)		
QoR-15 POD1, median (IQR)	102.5 (82–120) (*n*=122)	104.5 (86–121) (*n*=132)	−2.0 (−9.0 to 13.5)	0.39∗
QoR-15 POD4, median (IQR)	115.5 (96–129) (*n*=110)	115 (94–131) (*n*=128)	0.5 (−10.0 to 7.0)	0.78∗
QoR-15 POD7, median (IQR)	124 (107–137) (*n*=102)	124 (104–137) (*n*=126)	0.0 (−10 to 7.0)	0.56∗
Hospital consultation, *n* (%)	72 (48)	69 (45)	2.9 (−8.3% to 14.2%)	0.65†
Patient-reported recovery problems, *n* (%)	47 (32)	32 (21)	10.5% (0.6–20.3%)	0.05†
Hospital-recorded complication, *n* (%)	15 (10)	17 (11)	−1.1 (−8.1 to 5.8)	0.65†
Clavien–Dindo Grade I, *n* (%)	9 (6)	12 (8)		
Clavien–Dindo Grade II, *n* (%)	4 (3)	5 (3)		
Clavien–Dindo Grade III, *n* (%)	-	-		
Clavien–Dindo Grade IIIa, *n* (%)	1 (1)	-		
Clavien–Dindo Grade IIIb, *n* (%)	1 (1)	-		
Clavien–Dindo Grade Iva, *n* (%)	-	-		
Clavien–Dindo Grade Ivb, *n* (%)	-	-		
Clavien–Dindo Grade V, *n* (%)	-	-		
Satisfaction with care, median (IQR)	8.0 (7.0–10.0) (*n*=133)	8.0 (7.0–10.0) (*n*=140)	0.0 (0.0–0.5)	0.60∗
Trust in care, median (IQR)	8.0 (7.0–10.0) (*n*=133)	8.5 (7.0–10.0) (*n*=140)	0.5 (0.0–1.0)	0.16∗
Recommend hospital, median (IQR)	8.0 (7.0–10.0) (*n*=133)	9.0 (8.0–10.0) (*n*=140)	1.0 (0.0–2.0)	0.002∗

IQR, interquartile range; 95% CI, 95% confidence interval, POD, postoperative day; QoR-15, Quality of Recovery-15.

∗ Mann–Whitney *U*.

† χ^2^.

### Secondary outcome

QoR-15 and secondary outcomes at baseline and postoperative day 1 and 4, the number of hospital consultations, and the number of hospital recorded complications are presented in Table 2. There were no clinically relevant differences between study groups in secondary outcomes. Median difference in QoR-15 on POD1 was −2.0 (95% CI: −9.0 to 13.5; *P*=0.39), and on POD4, the median difference was 0.5 (95% CI: −10.0 to 7.0; *P*=0.78). More patients (47; 32%) in the smartphone group reported issues after discharge (pain, nausea, and concerns about wound care) compared with patients in the standard care group (32; 21%), with a proportional difference of 11% (95% CI: 0.6–20.3; *P*=0.05). Satisfaction with care and trust in the hospital were rated similarly by patients in both groups. For satisfaction, the median difference was 0.0 (95% CI: 0.0–0.5; *P*=0.60), and for trust in care, 0.5 (95% CI: 0.0–1.0; *P*=0.16). Recommending the hospital was rated higher in the standard care group, median 8 (IQR: 7.0–10.0), compared with the smartphone group, median 9.0 (IQR: 8.0–10.0), with a difference of 1.0 (95% CI: 0.0–2.0; *P*=0.002). *Post hoc*, we found a significant positive relationship between QoR-15 scores and patient satisfaction (correlation coefficient: 0.518; *P=<*0.001); see also Supplementary material.

## Discussion

This multicentre non-blinded randomised trial evaluated the effect of a smartphone application for postoperative follow-up on patient-reported quality of recovery after day care surgery. The smartphone application was studied in addition to standard care. Both groups received standard care, which included verbal and written discharge instructions. We observed no statistically significant nor clinically relevant differences between the smartphone group and standard care groups in patient-reported quality of recovery in day care surgery patients. When designing the trial, we deliberately selected postoperative day 7 as the primary endpoint to ensure that relevant adverse outcomes, such as pain, nausea, or complications, would not be missed, as these may occur beyond the immediate postoperative period. At the time of trial design, there were no evidence-based guidelines available to determine the optimal timing or frequency of follow-up after day case surgery. Our intention was to capture the full scope of patient recovery during the first postoperative week. Importantly, our secondary endpoints on days 1 and 4 also showed no differences between groups, which we believe further supports the robustness of our findings.

Although the smartphone group reported more recovery issues after discharge, the increased reporting did not result in enhanced outcomes. However, there is a difference in compliance to the QoR-15 which is lower in the smartphone group; a possible explanation is that patients who recorded their recovery in the application may have felt less need to also complete the QoR-15 questionnaire, leading to a lower response rate.

These results stand in contrast with previous studies reporting more positive outcomes for smartphone-based postoperative follow-up. For example, a study of 997 patients found significantly better values in seven of 24 Swedish Quality of Recovery (SwQoR) items on postoperative day 7 using a smartphone-based assessment.^33^ However, these findings were not based on a sum score, raising the possibility of chance findings due to multiple testing. The RecoverWell application, used in a breast cancer surgery trial, showed improved quality of recovery with app-assisted follow-up compared with in-person care, although it lacked integration with electronic medical records.^34^ The MServ app, limited to Android and in-hospital use, allowed thoracic and urogenital surgery patients to self-report pain over 48 h. It showed improved pain control and reduced opioid use at discharge.^35^ The Home to Stay app, tested in colorectal surgery patients, led to statistically significant improvements in satisfaction and well-being, aligned with the minimal clinically important difference for QoR-15, although again based on individual items rather than the total score.^36^ A systematic review of 27 studies concluded that eHealth interventions produced similar or better outcomes than face-to-face care, but most studies were of low to moderate quality and in early development.^10^

Our study has several limitations. First, there is a potential for selection bias owing to the inclusion of only Dutch-speaking smartphone users. Moreover, the smartphone-based approach may have favoured younger, more educated patients with a higher SES, which could limit the generalisability of our findings to patients with different language backgrounds or limited access to digital tools. To address this, we applied broad eligibility criteria, inviting all patients undergoing eligible surgery to participate, regardless of age, education level, or digital literacy. Patients were also offered assistance in installing and using the app to help reduce digital barriers. It should be noted that 260 out of 581 patients who were eligible declined to participate in the study. According to Dutch legislation, it is prohibited to collect any characteristics from these patients that might have been relevant to the acceptance of the application. Because we could not collect data on non-responders, a selection bias cannot be ruled out. To assess representativeness, we systematically collected patient characteristics, including age, gender, and SES. These characteristics were comparable between groups. Notably, SES in both groups was below the national Dutch average, reflecting the socioeconomic profile of the Amsterdam area where both hospital locations are situated.

Additionally, a number of patients had incomplete or inconsistently timed follow-up data, and feedback from medical staff was only available during office hours, which may have affected patient engagement and the timely management of symptoms. However, by implementing the intervention in a real-world hospital setting without strict exclusion criteria, we aimed to reflect routine clinical practice and strengthen the external validity of our findings.

In conclusion, adding a smartphone application for postoperative follow-up did not improve quality of recovery compared with standard care alone in day care surgical patients. This highlights the necessity of rigorous testing and careful implementation of digital health technologies to ensure they provide meaningful benefits. Although smartphone applications may have potential in postoperative care, their impact and integration into clinical practice require further investigation.

## Authors’ contributions

Contributed to conception and design, acquisition of data, analysis and interpretation of data; drafted the article and revised it critically for important intellectual content; gave final approval of the version to be published: BT, MG, SK, LV, CK

Contributed to acquisition of data, drafted the article revised it critically for important intellectual content, gave final approval of the version to be published; MvE, LvV, ER, MS

Agreed to be accountable for all aspects of the work, thereby ensuring that questions related to the accuracy or integrity of any part of the work are appropriately investigated and resolved: all authors

## Funding

This work was supported by, Stichting Internet Domeinregistratie Nederland fonds (SIDN fund). SIDN fund is a non-profit public benefit organization for Dutch internet domain registration (https://www.SIDNfonds.nl), and funded allocated time resources specifically for the researchers spent on writing the study protocol, conducting the research, analysing the data and preparing the manuscript. SIDN fund did not participate in any aspect of the study design, execution, data collection, analysis, decision to publish, or the preparation of the manuscript content. SIDN fund supports projects that contribute to a robust internet and digital applications for society.

## Declaration of interest

The authors declare that they have no conflicts of interest.
