# Supplementary material for: Discharge with a smartphone application for follow-up after day care surgery: a randomised controlled trial
Source: BJA Open. 2025 Sep 25;16:100489. doi: 10.1016/j.bjao.2025.100489 (PMC12509104; doi:10.1016/j.bjao.2025.100489)
Supplement: Multimedia Component 1 [file mmc1.docx]

Supplemental material 1

At the reviewer’s suggestion, we have explored the following correlations specifically for POD7, the primary endpoint: the correlation between the QoR-15 scores and patient satisfaction scores, and the correlation between QoR-15 scores in patients with and without complications.

For exploring the correlation between the QoR-15 scores and patient satisfaction we performed a Spearman rank correlation because the data are not normally distributed, the correlation coefficient is 0.518 (1-tailed, hypothesis is directional better satisfaction leads to better QoR-15) significance is <.001. Therefore, it can be concluded that there is a significant positive relationship between QoR-15 scores and satisfaction scores.

For exploring the correlation between the QoR-15 scores in patients with and without complications we performed a Spearman rank correlation because we explored a not normally distributed continuous variable (QoR-15 score) and a dichotomous variable (complication and no complications). We found a correlation coefficient -,089 (1 tailed, hypothesis is directional having complications leads to lower QoR-15) with a significance = 0,091. The sign of the correlation (positive or negative) depends on the coding of the dichotomous variable (complications = 1 & no complications = 0). Because R^2^ = (-.089)^2^, we can conclude that having complications accounts for 0.8% of the variability in QoR -15 scores; however, this is not significant with p 0,091. Therefore, it can be concluded that there is no statistical significant correlation between the QoR-15 and having postoperative complications.
